# High folate receptor expression is associated with aggressive features in prostate cancer with low prostate‐specific membrane antigen expression

**DOI:** 10.1002/bco2.70223

**Published:** 2026-05-21

**Authors:** Ashorne K. Mahenthiran, Burak Akgul, Mohammed Alshalalfa, Mark A. Green, Giovanna A. Giannico, Elai Davicioni, Daniel A. Sidhom, Michael O. Koch, Clinton D. Bahler

**Affiliations:** ^1^ Department of Urology Indiana University School of Medicine Indianapolis Indiana USA; ^2^ Department of Research and Development Veracyte Inc. South San Francisco California USA; ^3^ Department of Radiology and Imaging Sciences Indiana University School of Medicine Indianapolis Indiana USA; ^4^ Department of Pathology and Laboratory Medicine Indiana University School of Medicine Indianapolis Indiana USA

**Keywords:** folate receptor, genomics, prostate cancer, prostate‐specific membrane antigen, urologic oncology

## Abstract

**Objective:**

This work aimed to analyse the expression of and correlation between folate transporters FOLR1, FOLR2, SLC19A1 (reduced folate carrier, RFC), and FOLH1 (PSMA mRNA) in a large cohort of prostate cancer samples, with the goal of better understanding the nature of aggressive disease with low PSMA expression.

**Subjects and Methods:**

A total of 55 329 radical prostatectomy (RP) tumour specimens tested with the Decipher prostate genomic classifier (Veracyte, CA) were identified from the Decipher GRID database (NCT02609269). Transcriptome‐wide mRNA expression and baseline clinicopathologic data were retrieved. Logistic regression assessed associations between gene expression, pathological Gleason grade group (GG) 4–5, lymph node invasion (LNI), seminal vesicle invasion (SVI), and very high Decipher score (>0.85).

**Results:**

This cohort had a median age of 65 years and PSA of 4.9 ng/mL. FOLR1and FOLR2 showed no correlation with PSMA. High FOLR2 and RFC were associated with very high Decipher scores and GG4–5. In the lowest 10% PSMA group, high FOLR2 correlated with high Decipher scores, GG4–5, LNI, and SVI (all *p* < 0.001). Limitations include the retrospective design and the inability to distinguish FR‐β expression from immune cell infiltration.

**Conclusions:**

Our large‐scale study of transcriptome demonstrates FOLR2 gene expression is associated with aggressive prostate cancer in samples with low expression of PSMA. Future prediction of disease risk could be enhanced by using FOLR2 as a molecular target for identifying aggressive prostate cancer in cases of low PSMA.

## 1 INTRODUCTION

Most prostate cancers are adenocarcinoma.^1^ A key feature of this disease is prostate‐specific membrane antigen (PSMA), which is a transmembrane glycoprotein that generally demonstrates increased expression in prostate adenocarcinoma when compared to benign prostate tissue.^2^ As such, the subsequent implementation of the PSMA positron emission tomography (PET) scan has become commonplace for diagnostic confirmation of organ‐confined disease and for detection of the location(s) of recurrence after first‐line prostatectomy or radiation therapy.^3^ Prior investigation has also demonstrated that there is a positive correlation between traditional serum prostate‐specific antigen (PSA) levels, PSMA mRNA expression, and Gleason grade group (GG) when studied in radical prostatectomy specimens.^4^, ^5^

While aggressive prostate cancers do typically express higher levels of PSMA, there exists a unique subset of patients who do not follow this correlation.^6^ Specifically, neuroendocrine variant pathology has been associated with aggressive disease without a significant increase in PSA or PSMA.^7^, ^8^ Moreover, it has been demonstrated that patients with neuroendocrine variants have decreased expression of FOLH1 (PSMA), which is the gene responsible for PSMA encoding. The moderate correlation between FOLH1 expression and avidity on PSMA PET has previously been demonstrated by Weiner et al. and Pramod et al.^9^, ^10^ These patients are less responsive to hormonal treatment and, as such, are at increased mortality risk.^7^ Since this phenotype of prostate cancer is often harder to accurately detect with conventional modalities such as PSA or PET PSMA, there exists a need to develop novel strategies for diagnosis.

The folate receptor (FR) is a cell membrane target for high‐affinity binding, which serves as a target for tumour‐selective delivery of diagnostic and therapeutic agents.^11^ Prior studies have demonstrated that circulating tumour cells in prostate cancer patients have overexpression of FR, and that this was consistent regardless of the patient's PSA level.^12^ As such, it has been hypothesized that FRs, particularly FR‐α (FOLR1) and FR‐β (FOLR2), or their transporter, SLC19A1‐reduced folate carrier protein (RFC), may serve as alternate diagnostic markers for prostate cancer patients. This study aims to characterize the expression of FOLR1, FOLR2, RFC, and PSMA in a large cohort of prostate cancer patients.

## 2 SUBJECTS AND METHODS

### 2.1 Patient population

Prospectively collected 55 329 radical prostatectomy (RP) specimens tested with the Decipher prostate genomic classifier (GC) between 2016 and 2023 were retrieved from the Decipher GRID registry (NCT02609269).^11^ Transcriptome‐wide mRNA expression data of 46 000 coding and non‐coding genes, as well as baseline clinical and pathologic factors (age, preoperative PSA, GG 4–5 at time of radical prostatectomy, lymph node invasion [LNI], seminal vesicle invasion [SVI]), were retrieved. Clinical outcomes from follow‐up data were not available for this analysis. Patient data was de‐identified in accordance with the Safe Harbour method described in the HIPAA Privacy Rule 45 CFR 164.514(b) and (c) (Veracyte, Inc., San Diego, CA) before analysis.

### 2.2 Expression data and pathological variables

The Decipher genomic classifier (GC) utilizes 22 genes to generate a risk score for clinical use (Veracyte Inc., San Diego, CA, USA). The GC risk score is described pre‐defined cutoffs of <0.45, 0.45–0.6, 0.6–0.85, and >0.85 to stratify patients by risk of adverse prostate cancer pathology. We elected to use >0.85 as the numerical threshold for very high GC (VHD) score, as scores above this have demonstrated adverse clinical outcomes.^13^, ^14^ Expression of FOLH1, FOLR1, FOLR2, and RFC was extracted for all samples. Of note, FOLH1 and PSMA were used interchangeably in this analysis, with the understanding that PSMA RNA is transcribed from the FOLH1 gene to direct PSMA protein production.

### 2.3 Statistical analysis and outcomes

The statistical significance of differences in pathological characteristics across categorized gene expression was determined via the chi‐squared test. Logistic regression was used to evaluate associations between expression of each of the different genes of study (top 10% [high] versus bottom 90% [low]), GG 4–5, LNI, SVI, and VHD score (>0.85). Since this cohort lacks clinical outcomes for the patients, VHD score was used as the primary surrogate clinical endpoint. We also performed several subanalyses to directly compare odds of adverse pathology directly between each gene of study and to also estimate the proportion of neuroendocrine‐like cancers based on levels of expression of each of the genes of study. All analyses were performed using R version 4.2.

## 3 RESULTS

### 3.1 Baseline characteristics

The cohort, consisting of 55 329 radical prostatectomy cases, had a median age of 65 years (IQR 60–69) and a median preoperative PSA level of 4.9 ng/mL (IQR 0.22–8). Patient characteristics are summarized in Table 1. The distribution according to Gleason score was as follows: 4.3% for GG1, 42.4% for GG2, 30.9% for GG3, and 22.2% for GG4–5. Based on the Decipher genomic classification, 23.5% of the samples were categorized as having a VHD score. Pathological evaluation revealed extraprostatic extension in 55.6% of cases, lymph node invasion in 4.6%, and seminal vesicle invasion in 19.1%, indicating a cohort with a broad spectrum of disease aggressiveness and a notable proportion of patients exhibiting adverse pathological and genomic features.

**TABLE 1:** Clinicopathological and genomic characteristics of the study cohort.

Parameter	GRID RP
Total	55 329
Median age (years) [IQR]	65 [60–69]
Median PSA (ng/mL) [IQR]	4.9 [0.22–8]
Pathology grade group	
1	2398 (4.3%)
2	23 488 (42.4%)
3	17 106 (30.9%)
4	4279 (7.7%)
5	7999 (14.5%)
Decipher risk groups	
Low/intermediate (<0.6)	26 719 (48.3%)
High (0.6–0.85)	15 600 (28.2%)
Very high (>0.85)	13 010 (23.5%)
Extraprostatic extension	
Positive	30 747 (55.6%)
Negative	24 578 (44.4%)
Lymph node invasion	
Positive	2553 (4.6%)
Negative	52 774 (95.3%)
Seminal vesicle invasion	
Positive	10 580 (19.1%)
Negative	44 745 (80.8%)

### 3.2 Comparison of FR expression within the cohort

**FIGURE 1:**
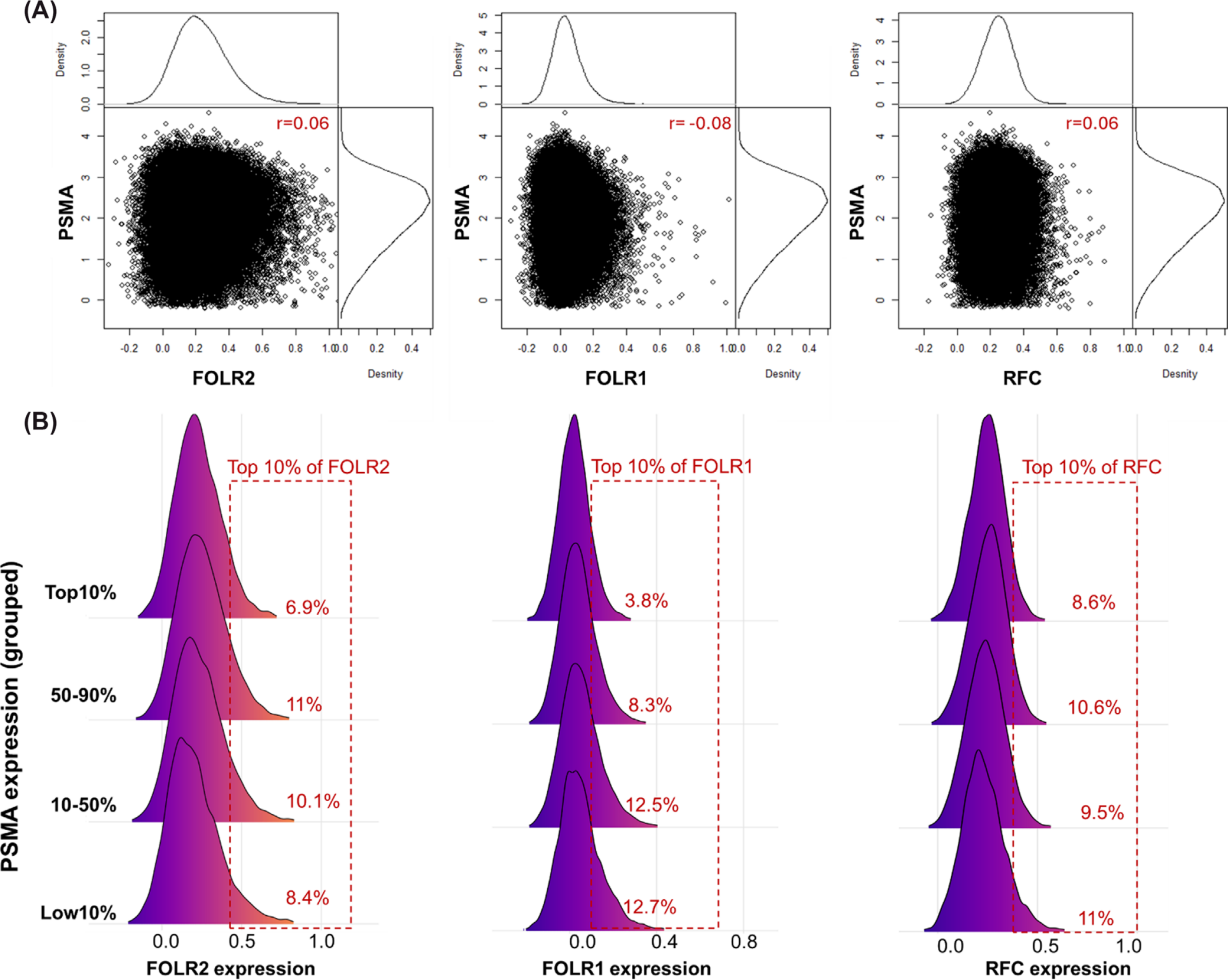
Evaluating correlation between expression of PSMA and folate‐related genes. (A) Scatter‐density plots illustrate pairwise correlations between FOLH1 (PSMA) and the folate‐transporter genes FOLR2 (FR‐β), FOLR1 (FR‐α), and SLC19A1 (RFC) across all samples. Correlation coefficients (*r*) are displayed in red, highlighting overall weak relationships between PSMA and each folate‐related gene. (B) Box and density plots visualize the distribution of FOLR2 (FR‐β), FOLR1 (FR‐α), and SLC19A1 (RFC) expression across FOLH1 (PSMA) expression ranges: low (bottom 10%), intermediate (10%–50% and 50%–90%), and high (top 10%). Expression patterns are shown to illustrate the relative variation of folate‐transporter activity across differing PSMA levels. Boxes show the percentage (%) of patients among the top 10% of folate transporter genes across different levels of PSMA expression highlighting high folate expression is enriched in low PSMA samples.

Across the transcriptomic dataset, folate transporter genes (FOLR1, FOLR2, and RFC) exhibited overall lower expression levels compared to PSMA. Among these, FOLR2, which encodes FR‐β, was expressed at significantly higher levels than SLC19A1 (encoding RFC) and FOLR1 (encoding FR‐α) (*p* < 0.001, Figure S1). Correlation analysis revealed weak overall correlation between PSMA and the other folate‐related genes (*r* = 0.06, Figure 1A). In contrast, the expression levels among the folate transporter genes themselves showed weak to moderate positive correlations (Figure S2). Additionally, in samples with low PSMA expression (bottom 10%), a slight increase in FOLR1 and FOLR2 expression was observed (Figure 1B).

### 3.3 Association of FR/RFC and PSMA expression with aggressive disease

**FIGURE 2:**
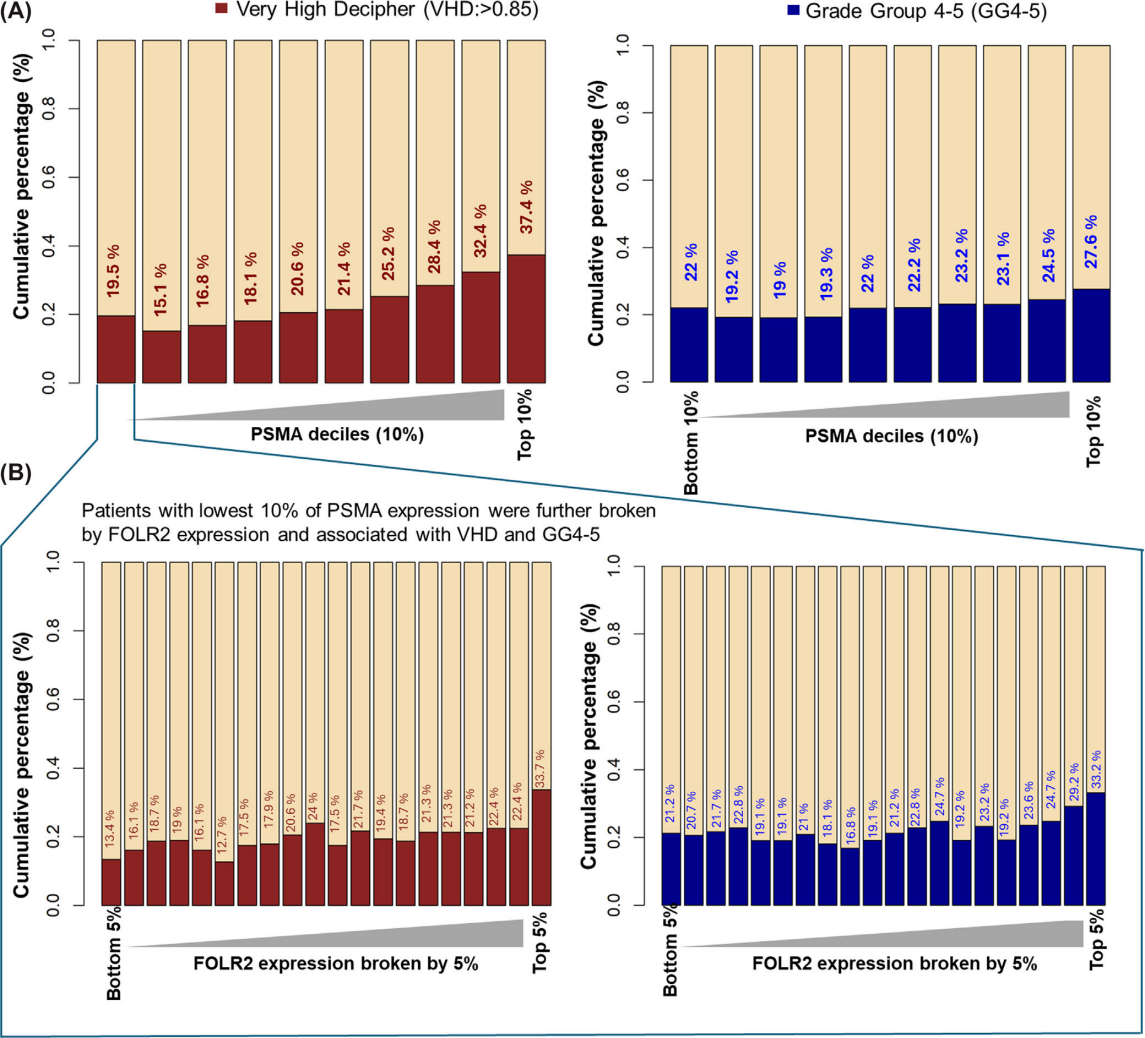
Association between PSMA expression and aggressive prostate cancer features including subset of FOLR2 expression in low PSMA samples. (A) Distribution plots depict the relationship between increasing FOLH1 (PSMA) expression levels and markers of aggressive disease, including very high decipher (VHD) scores and Gleason grade group (GG) 4–5 disease. (B) Corresponding plots for FOLR2 (FR‐β) with samples with lowest 10% of PSMA expression highlighting the distribution of high‐risk pathological and genomic features in samples with high FOLR2 expression.

There were several notable associations in this cohort between PSMA, FOLR2, and RFC. Overall, samples with the highest amount of PSMA expression were associated with higher rates of VHD scores and GG 4–5 (Figure 2A). However, in samples with the lowest 10% of PSMA expression – those with the highest FOLR2 expression were associated with VHD scores and GG 4–5 (Figure 2B). Similar to increasing PSMA expression, a higher percentage of VHD scores and GG 4–5 appeared to be present with increasing FOLR2 and RFC expression (Figure S3).

**FIGURE 3:**
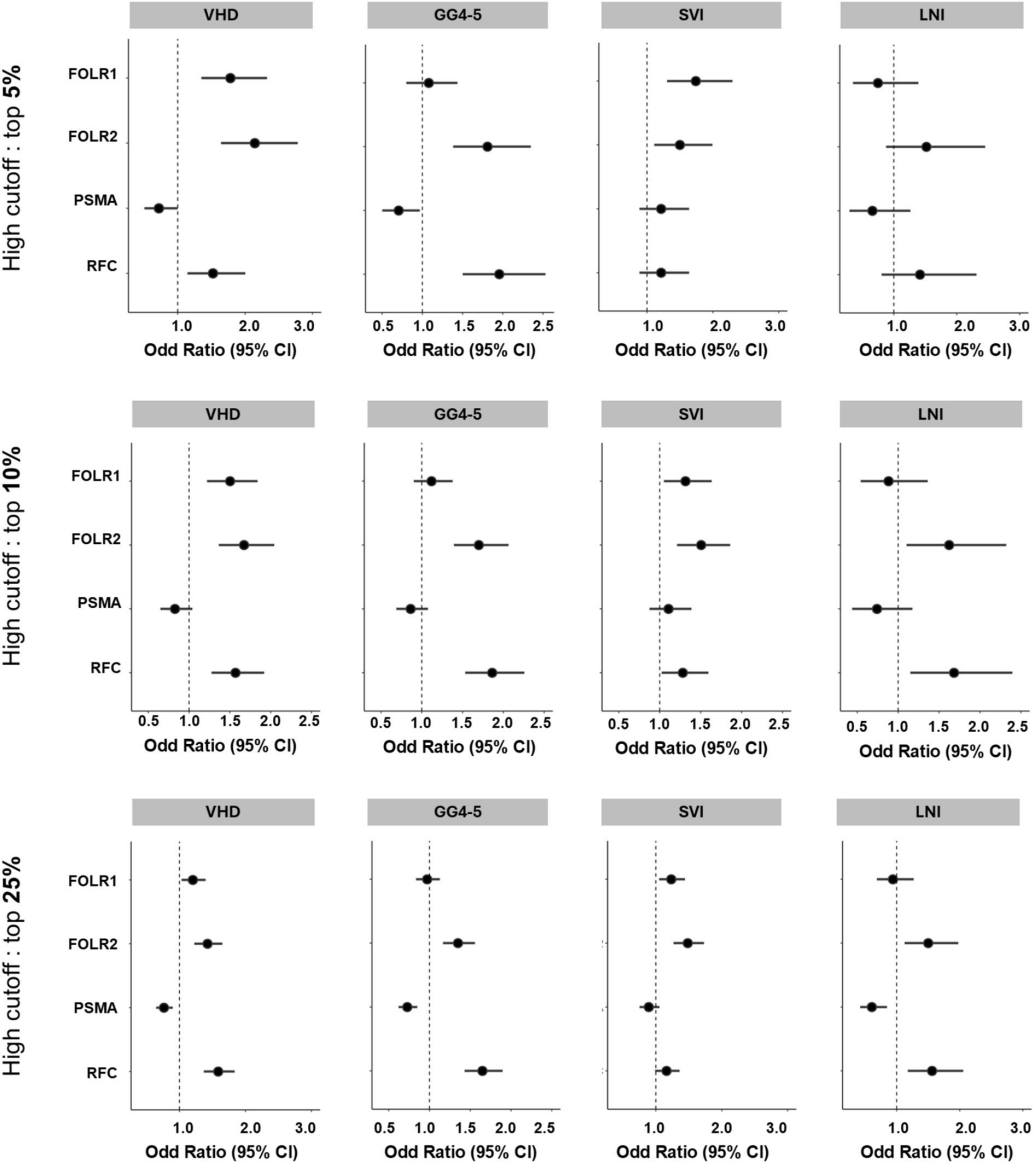
Association of high folate transporter gene expression (stratified as top 5%, top 10%, and top 25%) with adverse molecular and pathological features in low PSMA samples . Forest plots display the odds ratios for adverse pathological endpoints [very high decipher (VHD) scores, Gleason grade group (GG) 4–5, lymph node invasion (LNI), and seminal vesicle invasion (SVI)] comparing tumours with high FOLR2 (FR‐β) or SLC19A1 (RFC) expression (top 5%, top 10%, and top 25%) to all other cases within the low FOLH1 (PSMA) group.

We next evaluated the odds ratios of high gene expression to predict different endpoints in the low PSMA group. Both FOLR2 and RFC were more associated with the pathological endpoints GG4–5, LNI, and SVI (*p* < 0.001) when top 10% was used as the cutoff for high gene expression, and this pattern of association was maintained when using top 5% and top 25% (Figure 3). To further evaluate this association, we assessed the odds ratio of predicting VHD, LNI, SVI, and GG4–5 across all deciles of PSMA. In the low PSMA group, RFC was most predictive of GG4–5 and LNI, while FOLR2 was most predictive of VHD. FOLR1 showed a similar pattern of association to FOLR2, but only associated with VHD in the lowest 20% of PSMA expression (Figure S4).

To further shed biological insights into this association of high FOLR1/2 with VHD in low PSMA groups, we associated the expression of these genes with neuroendocrine‐like tumours based on two previously developed gene expression signatures.^15^, ^16^ The samples were annotated as NE‐like if they were called NE‐like by either of the signatures. Overall, we observed that 2.93% of the samples were called NE‐like. However, this percentage increased almost threefold in patients with low PSMA (bottom 10%) or high FOLR1 or 2 (top 10%) (Figure S5). The percentage of NE‐like was increased to 16% in samples with low PSMA (low 10%) and high FOLR1/2 (top 10%) and further increased to 27%–32% for samples falling within the top 5% of FOLR1/2 and lowest 5% of PSMA. This highlights the potential clinical utility of FOLR1/2 as targets for imaging modalities in NE‐like tumours.

## 4 DISCUSSION

In this large‐scale transcriptomic analysis encompassing over 55 000 radical prostatectomy specimens, we identified a distinct subset of prostate cancers that demonstrate aggressive clinical and genomic characteristics despite low PSMA expression. PSMA is highly expressed in over 90% of prostate cancer lesions.^2^, ^3^ Therefore, it is an important molecular target for both therapy and imaging to determine the location and extent of disease. However, the degree and homogeneity of expression vary among patients. Reduced PSMA expression, particularly in prostate cancers with androgen receptor‐independent or neuroendocrine differentiation, is associated with poor response to PSMA‐targeted PET imaging and radioligand therapies.^7^, ^17^ Clinically, approximately 30% of patients with metastatic castration‐resistant prostate cancer also have PSMA‐negative but FDG‐positive metastatic lesions.^18^ This suggests that PSMA‐based imaging and treatment approaches may be inadequate in certain subgroups.

Serum PSA levels, often an easily applicable first‐line marker, also exhibit biological heterogeneity that may underestimate disease aggressiveness in this patient group. Clinically, high‐grade prostate cancer with low PSA levels is characterized by poor prognosis and neuroendocrine dedifferentiation.^7^, ^19^ In this subtype, decreased androgen receptor signalling and increased neuroendocrine markers suggest a biological phenotype that is poorly responsive to hormonal therapy.^8^, ^18^ Previous studies have shown that diagnosis in this patient group is often delayed and that this delay is associated with an increased risk of mortality.^8^ This may be due to the growing use of PET PSMA for surveillance of localized prostate cancer, and as such, patients with minimal PSMA expression are deemed lower risk for clinically relevant disease.^20^ In this patient group with low PSMA expression, genetic analyses on biopsy or surgically resected tissues indicate that the use of complementary imaging agents directed against non‐PSMA molecular targets with independent genomic risk markers allows for a more accurate and comprehensive assessment of disease burden.^3^, ^21^

It is remarkable that in our study, among over 55 000 radical prostatectomy specimens, more than half of the patients had a Gleason score ≥4 + 3, yet the mean PSA level was only 4.9 ng/mL. This finding supports the existence of a subgroup of aggressive prostate cancers with high‐grade disease but low PSA production. Analysis of FOLR1, FOLR2, RFC, and PSMA expression profiles in this cohort sheds light on the molecular heterogeneity of prostate cancer. Our findings indicate that alternative folate transporter pathways, such as FOLR2 and, in part, RFC, are significantly increased in tumours with low PSMA expression. This increase is closely associated with poor prognostic features such as GG 4–5, seminal vesicle invasion, and high Decipher scores. PSMA was found to be the marker with the highest overall expression level; however, FR‐β expression was higher than FR‐α and RFC and did not correlate with PSMA levels. Increased FR‐β levels were significantly associated with aggressive tumour behaviour, particularly in patients with PSMA expression in the lowest 10%.

Previous studies have shown that folate receptor overexpression is not limited to prostate cancer, but rather is a common molecular feature of many malignancies.^22^, ^23^ FOLR1 is generally expressed in malignant cells of epithelial origin, while FOLR2 is expressed in tumour‐associated macrophages (TAMs) and contributes to tumour progression.^24^ RFC is a folate transporter protein that is widely but non‐selectively found in normal tissues.^25^ FOLR1 and FOLR2 have previously been associated with ovarian, breast, lung, and pancreatic cancers; furthermore, the clinical relevance of FR has been highlighted by its expression in circulating tumour cells in prostate cancer patients without overt PSA elevation.^19^, ^20^, ^22^

Like PSMA, the folate receptor mediates the selective uptake of targeted agents into tumour cells via endocytosis following ligand binding. This biological mechanism has been successfully applied to many solid tumours through the development of technetium‐99m and gallium‐68‐based tracers (e.g., 99mTc‐etarfolatide and 68Ga‐deferoxamine‐folate) and folate‐linked drug conjugates.^26^ These agents have demonstrated good tumour selectivity and safety profiles in early‐stage clinical studies in ovarian, lung, and renal cancers. More recently, the development of novel FR‐based PET tracers with increased in vivo sensitivity has further accelerated progress in this field. Among the therapeutic approaches, vintafolide (a folate molecule conjugated with vinblastine) was evaluated in the PROCEED and TARGET studies in ovarian and lung cancers, respectively, but failed to provide a survival advantage.^27^, ^28^ Of note, while these studies primarily focused on FR‐α targeted therapies, our data suggest that FR‐ß may be a more clinically relevant subtype of prostate cancer.^24^, ^27^, ^28^

The interpretation of our findings must be viewed within the context of inherent limitations. First, the analyses were based on ex vivo mRNA expression profiles obtained from radical prostatectomy specimens, and these results may not fully reflect the in vivo FR‐positivity levels observed in clinical diagnoses. Furthermore, it remains unclear whether FOLR2 expression originates from tumour epithelial cells or tumour‐associated macrophage infiltration, limiting the biological interpretation of our findings. Second, because the study was a retrospective cohort analysis using transcriptomic data, long‐term clinical outcomes such as biochemical recurrence, metastasis, or survival are not available. Third, patient‐related factors such as comorbidities, prior treatments, and demographic differences were not included in the dataset, and these variables may have influenced the gene expression results. Finally, the study was conducted solely at the gene expression level, and protein‐level or functional validation (e.g., immunohistochemistry or cell line experiments) was not performed; therefore, the increase in FOLR2 expression should be considered a correlational, not causal, finding.

Clinically, the marked enrichment of NE‐like tumours in samples with low PSMA avidity and high FOLR1/2 expression highlights a significant diagnostic gap, as this biologically aggressive subtype frequently evades both PSA‐ and PSMA‐based detection. Our findings indicate that FR‐β represents a promising future diagnostic and prognostic target for aggressive prostate cancer patients who remain undetected by these conventional methods.

## 5 CONCLUSIONS

This large‐scale transcriptomic analysis demonstrated that high FOLR2 expression, particularly in tumours with low PSMA expression, is independently associated with adverse pathological and genomic features, including GG 4–5, seminal vesicle, and lymph node invasion, and high Decipher scores. Within samples with low PSMA, high FOLR2 is highly indicative of NE‐like biology. These findings indicate that FOLR2 may serve as a complementary biomarker for the diagnosis and risk stratification of biologically aggressive prostate cancers with low PSMA expression and may provide a foundation for developing non‐PSMA–based imaging and targeted therapeutic approaches. However, the retrospective design of the study and the absence of protein‐level validation underscore the need for prospective confirmatory studies to identify FR‐positive prostate cancer using appropriate imaging modalities and to develop therapeutic strategies targeting the folate receptor. Developing alternative molecular diagnostic approaches for the early detection of this aggressive prostate cancer subtype is crucial to prevent diagnostic delays and the associated increased risk of mortality.

## AUTHOR CONTRIBUTIONS

**Ashorne K. Mahenthiran:** Conceptualization; writing—original draft; writing—review and editing. **Burak Akgul:** Conceptualization; writing—original draft; writing—review and editing. **Mohammed Alshalalfa:** Conceptualization; investigation; formal analysis; writing—review and editing. **Mark A. Green:** Writing—review and editing; supervision. **Giovanna A. Giannico:** Writing—review and editing; supervision. **Elai Davicioni:** Investigation; supervision. **Daniel A. Sidhom:** Writing—original draft; writing—review and editing. **Michael O. Koch:** Writing—review and editing; supervision. **Clinton D. Bahler:** Conceptualization; supervision; funding acquisition.

## CONFLICT OF INTEREST STATEMENT

Mohammed Alshalalfa and Elai Davicioni are employees of Veracyte Inc. The other authors have no financial interests to disclose.

## Supporting information


**Figure S1.** Comparison of Overall Expression of PSMA and Folate‐Related Genes. Ridge density distribution plots display the expression patterns of PSMA (FOLH1), FOLR2 (FR‐β), RFC (SLC19A1), and FOLR1 (FR‐α) across all samples. Colour‐gradient maps illustrate relative gene expression intensity, ranging from low (purple) to high (yellow). PSMA (FOLH1) demonstrated the highest overall expression within the cohort, while FOLR2 (FR‐β) represented the predominant folate‐receptor subtype. B. Ridge density plot focused on FOLR1 and FOLR2 showing FOLR2 has higher expression.
**Figure S2.** Evaluating Correlation Between Expression of Folate‐Related Genes Scatter‐density plots illustrate pairwise correlations between each of the folate‐transporter genes themselves – including FOLR2 (FR‐β), FOLR1 (FR‐α), and SLC19A1 (RFC) across all samples. Correlation coefficients ® are displayed in red, highlighting weak to moderate positive correlations between each of the folate transporter genes.
**Figure S3.** Evaluating Percentage of Aggressive Prostate Cancer Features by Increasing PSMA, FOLR2, FOLR1 and RFC Expression Stratified by 5% Increments. Distribution plots depict the relationship between increasing FOLH1 (PSMA), FOLR2 (FR‐β), SLC19A1 (RFC), FOLR1 (FR‐α) expression and markers of aggressive disease, including Very High Decipher (VHD) scores and Gleason Grade Group (GG) 4–5 disease.
**Figure S4.** Association of High FOLR2, RFC, FOLR1 expression (Top 10%) with Aggressive Prostate Cancer Features Across Increasing Ranges of PSMA Expression Forest plots present the odds ratios (95% CI) for very high Decipher (VHD) scores (>0.85), Gleason Grade Group (GG) 4–5 disease, lymph node invasion (LNI), and seminal vesicle invasion (SVI) across deciles of FOLH1 (PSMA) expression, comparing tumours with high (top 10%) FOLR2 (FR‐β), SLC19A1 (RFC), and FOLR1 (FR‐α) expression to all samples.
**Figure S5.** Association of Varying PSMA, FOLR1/2, and RFC Gene Expression with Percentage of Neuroendocrine (NE)‐like Cancers.

## References

[1] Leslie SW , Soon‐Sutton TL , Skelton WP . Prostate Cancer. StatPearls [Internet] 2024. 29261872

[2] Donin NM , Reiter RE . Why targeting PSMA is a game changer in the management of prostate cancer. J Nucl Med. 2018;59(2):177–182. 10.2967/jnumed.117.191874 28986509 PMC6910619

[3] Calais J , Czernin J . PSMA expression assessed by PET imaging is a required biomarker for selecting patients for any PSMA‐targeted therapy. J Nucl Med. 2021;62(11):1489–1491. 10.2967/jnumed.121.263159 34725231 PMC8612346

[4] Cheng J , Adhami M , Pham T , Nadebaum DP , Baring A , Paul E , et al. Does total lesion prostate‐specific membrane antigen (PSMA) activity on (68)Ga‐PSMA PET/CT correlate with PSA and prostatectomy histopathological/clinical outcomes in patients with localised primary prostate cancer? BJUI Compass. 2025;6(4):e70015. 10.1002/bco2.70015 40241844 PMC12000823

[5] Rosales JJ , Antar VB , Mínguez F , Betech Antar V , Pareja F , Guillén F , et al. Comparison of staging using [^68^Ga]Ga‐PSMA‐11 PET/CT and histopathological results in intermediate‐ and high‐risk prostate cancer patients treated with radical prostatectomy and pelvic lymph node dissection. Rev Esp Med Nucl Imagen Mol (Engl Ed). 2025;44(2):500076. 10.1016/j.remnie.2024.500076 39477086

[6] Sokoloff MH , Yang XJ , Fumo M , Mhoon D , Brendler CB . Characterizing prostatic adenocarcinomas in men with a serum prostate specific antigen level of <4.0 ng/mL. BJU Int. 2004;93(4):499–502. 10.1111/j.1464-410X.2003.04657.x 15008717

[7] Bakht MK , Derecichei I , Li Y , Ferraiuolo RM , Dunning M , Oh SW , et al. Neuroendocrine differentiation of prostate cancer leads to PSMA suppression. Endocr Relat Cancer. 2018;26(2):131–146. 10.1530/ERC-18-0226 30400059

[8] Mahal BA , Yang DD , Wang NQ , Alshalalfa M , Davicioni E , Choeurng V , et al. Clinical and genomic characterization of low‐prostate‐specific antigen, high‐grade prostate cancer. Eur Urol. 2018;74(2):146–154. 10.1016/j.eururo.2018.01.043 29478736 PMC6615042

[9] Weiner AB , Agrawal R , Wang NK , Sonni I , Li EV , Arbet J , et al. Molecular hallmarks of prostate‐specific membrane antigen in treatment‐naïve prostate cancer. Eur Urol. 2024;86(6):579–587. 10.1016/j.eururo.2024.09.005 39294048 PMC11637967

[10] Pramod N , Katral S , Trostel S , Turkbey B , Choyke PL , Lindenberg L , et al. IP05‐10 comparison of 18F‐DCFPyL PSMA PET/CT uptake to PSMA protein expression in high‐risk prostate tumors from the NCI Prostate Moonshot study. J Urol. 2025;213(5S):e296. 10.1097/01.JU.0001109816.50355.3c.10

[11] Elnakat H , Ratnam M . Distribution, functionality and gene regulation of folate receptor isoforms: implications in targeted therapy. Adv Drug Deliv Rev. 2004;56(8):1067–1084. 10.1016/j.addr.2004.01.001 15094207

[12] Lian S , Yang L , Feng Q , Wang P , Wang Y , Li Z . Folate‐receptor positive circulating tumor cell is a potential diagnostic marker of prostate cancer. Front Oncol. 2021;11:708214. 10.3389/fonc.2021.708214 34692484 PMC8531518

[13] Sharma R , Moschovas MC , Bhatt SKR , Saikali S , Ozawa Y , Sandri M , et al. Prognostic implications of very high decipher scores in prostate cancer: towards a refined genomic risk classification. Eur Urol Oncol. 2026;9(1):72–79. 10.1016/j.euo.2025.09.009 41068038

[14] Handa N , Li EV , Michael J , Proudfoot JA , Weiner AB , Alam R , et al. Prevalence of potential candidates for targeted therapies according to treatment‐related transcriptomic signatures among 140 548 patients with nonmetastatic prostate cancer. Eur Urol Oncol. 2025;8(4):1050–1058. 10.1016/j.euo.2025.04.022 40350343

[15] Kumar A , Coleman I , Morrissey C , Zhang X , True LD , Gulati R , et al. Substantial interindividual and limited intraindividual genomic diversity among tumors from men with metastatic prostate cancer. Nat Med. 2016;22(4):369–378. 10.1038/nm.4053 26928463 PMC5045679

[16] Alshalalfa M , Liu Y , Wyatt AW , Gibb EA , Tsai HK , Erho N , et al. Characterization of transcriptomic signature of primary prostate cancer analogous to prostatic small cell neuroendocrine carcinoma. Int J Cancer. 2019;145(12):3453–3461. 10.1002/ijc.32430 31125117 PMC6852174

[17] Epstein JI , Amin MB , Beltran H , Lotan TL , Mosquera JM , Reuter VE , et al. Proposed morphologic classification of prostate cancer with neuroendocrine differentiation. Am J Surg Pathol. 2014;38(6):756–767. 10.1097/PAS.0000000000000208 24705311 PMC4112087

[18] Kim J , Coetzee GA . Prostate specific antigen gene regulation by androgen receptor. J Cell Biochem. 2004;93(2):233–241. 10.1002/jcb.20228 15368351

[19] Tan HL , Sood A , Rahimi HA , Wang W , Gupta N , Hicks J , et al. Rb loss is characteristic of prostatic small cell neuroendocrine carcinoma. Clin Cancer Res. 2014;20(4):890–903. 10.1158/1078-0432.CCR-13-1982 24323898 PMC3931005

[20] Liu J , Santucci J , Woon DTS , Catterwell R , Perera M , Murphy DG , et al. A systematic review on prostate‐specific membrane antigen positron emission tomography (PSMA PET) evaluating localized low‐ to intermediate‐risk prostate cancer: a tool to improve risk stratification for active surveillance? Life (Basel). 2024;14(1):76. 10.3390/life14010076 38255691 PMC10817570

[21] Ross AE , Johnson MH , Yousefi K , Davicioni E , Netto GJ , Marchionni L , et al. Tissue‐based genomics augments post‐prostatectomy risk stratification in a natural history cohort of intermediate‐ and high‐risk men. Eur Urol. 2016;69(1):157–165. 10.1016/j.eururo.2015.05.042 26058959

[22] Matherly LH , Hou Z . Structure and function of the reduced folate carrier a paradigm of a major facilitator superfamily mammalian nutrient transporter. Vitam Horm. 2008;79:145–184. 10.1016/S0083-6729(08)00405-6 18804694 PMC3806226

[23] Carll J , Shi W , Perera M , Lawrentschuk N , Chengodu T , Woon D . Guideline of guidelines: PSMA PET in staging newly diagnosed intermediate‐risk prostate cancer. BJU Int. 2025;136(5):800–804. 10.1111/bju.16872 40704877 PMC12522526

[24] Scaranti M , Cojocaru E , Banerjee S , Banerji U . Exploiting the folate receptor alpha in oncology. Nat Rev Clin Oncol. 2020;17(6):349–359. 32152484 10.1038/s41571-020-0339-5

[25] Shen J , Hu Y , Putt KS , Singhal S , Han H , Visscher DW , et al. Assessment of folate receptor alpha and beta expression in selection of lung and pancreatic cancer patients for receptor targeted therapies. Oncotarget. 2018;9(4):4485–4495. 10.18632/oncotarget.23321 29435118 PMC5796989

[26] Brand C , Longo VA , Groaning M , Weber WA , Reiner T . Development of a new folate‐derived Ga‐68‐based PET imaging agent. Mol Imaging Biol. 2017;19(5):754–761. 10.1007/s11307-017-1049-y 28194631 PMC5554734

[27] Vergote I , Leamon CP . Vintafolide: a novel targeted therapy for the treatment of folate receptor expressing tumors. Ther Adv Med Oncol. 2015;7(4):206–218. 10.1177/1758834015584763 26136852 PMC4480526

[28] Naumann RW , Coleman RL , Burger RA , Sausville EA , Kutarska E , Ghamande SA , et al. PRECEDENT: a randomized phase II trial comparing vintafolide (EC145) and pegylated liposomal doxorubicin (PLD) in combination versus PLD alone in patients with platinum‐resistant ovarian cancer. J Clin Oncol. 2013;31(35):4400–4406. 10.1200/JCO.2013.49.7685 24127448

