# Supplementary material for: High folate receptor expression is associated with aggressive features in prostate cancer with low prostate‐specific membrane antigen expression
Source: BJUI Compass. 2026 May 21;7(5):e70223. doi: 10.1002/bco2.70223 (PMC13239787; doi:10.1002/bco2.70223)

Figure S1. Comparison of Overall Expression of PSMA and Folate-Related Genes

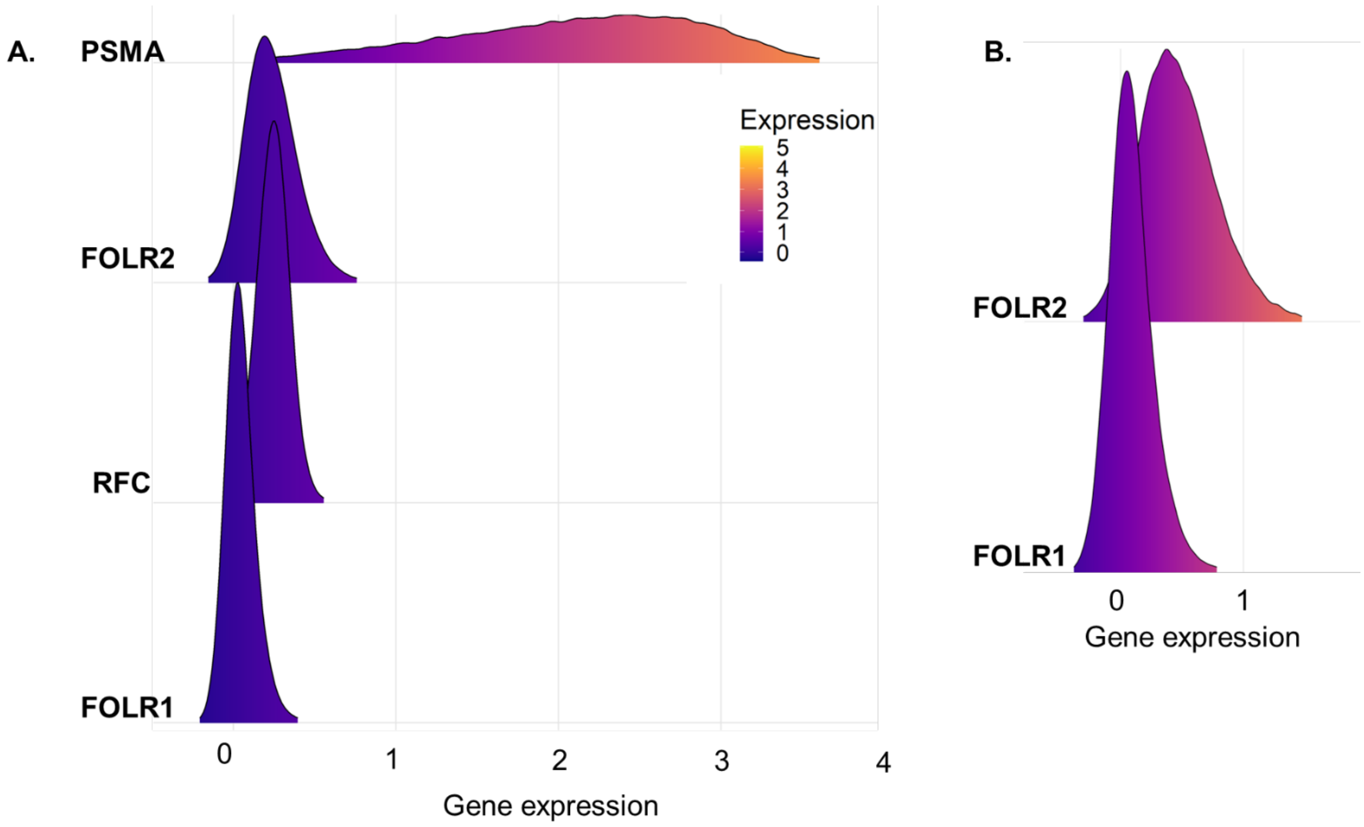

A. Ridge density distribution plots display the expression patterns of PSMA (FOLH1), FOLR2 (FR-β), RFC (SLC19A1), and FOLR1 (FR-α) across all samples. Color-gradient maps illustrate relative gene expression intensity, ranging from low (purple) to high (yellow). PSMA (FOLH1) demonstrated the highest overall expression within the cohort, while FOLR2 (FR-β) represented the predominant folate-receptor subtype.

B. Ridge density plot focused on FOLR1 and FOLR2 showing FOLR2 has higher expression.

Figure S2. Evaluating Correlation Between Expression of Folate-Related Genes

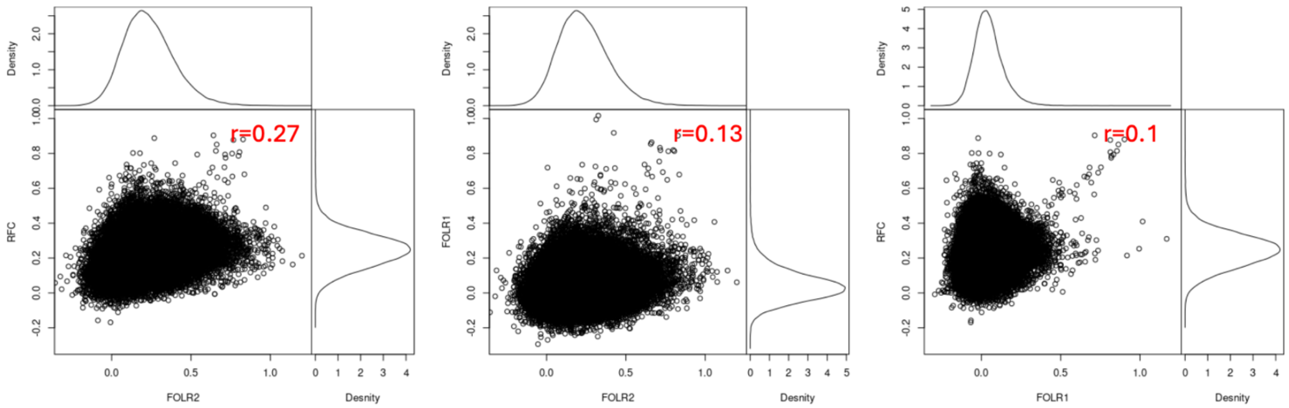

Scatter-density plots illustrate pairwise correlations between each of the folate-transporter genes themselves – including FOLR2 (FR-β), FOLR1 (FR-α), and SLC19A1 (RFC) across all samples. Correlation coefficients ® are displayed in red, highlighting weak to moderate positive correlations between each of the folate transporter genes.

Figure S3. Evaluating Percentage of Aggressive Prostate Cancer Features by Increasing PSMA, FOLR2, FOLR1 and RFC Expression Stratified by 5% Increments

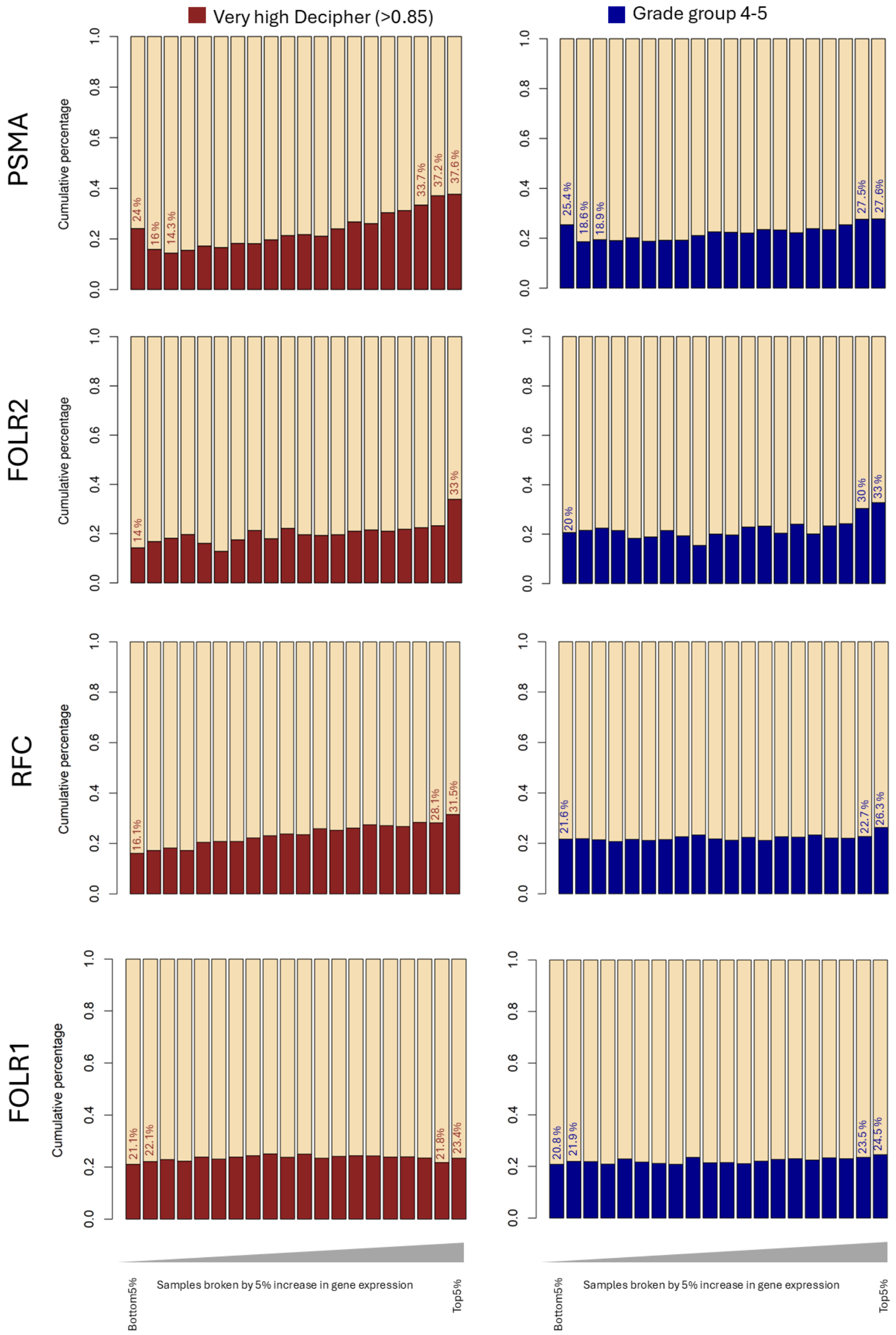

Distribution plots depict the relationship between increasing FOLH1 (PSMA), FOLR2 (FR-β), SLC19A1 (RFC), FOLR1 (FR-α) expression and markers of aggressive disease, including Very High Decipher (VHD) scores and Gleason Grade Group (GG) 4–5 disease.

Figure S4. Association of High FOLR2, RFC, FOLR1 expression (Top 10%) with Aggressive Prostate Cancer Features Across Increasing Ranges of PSMA Expression

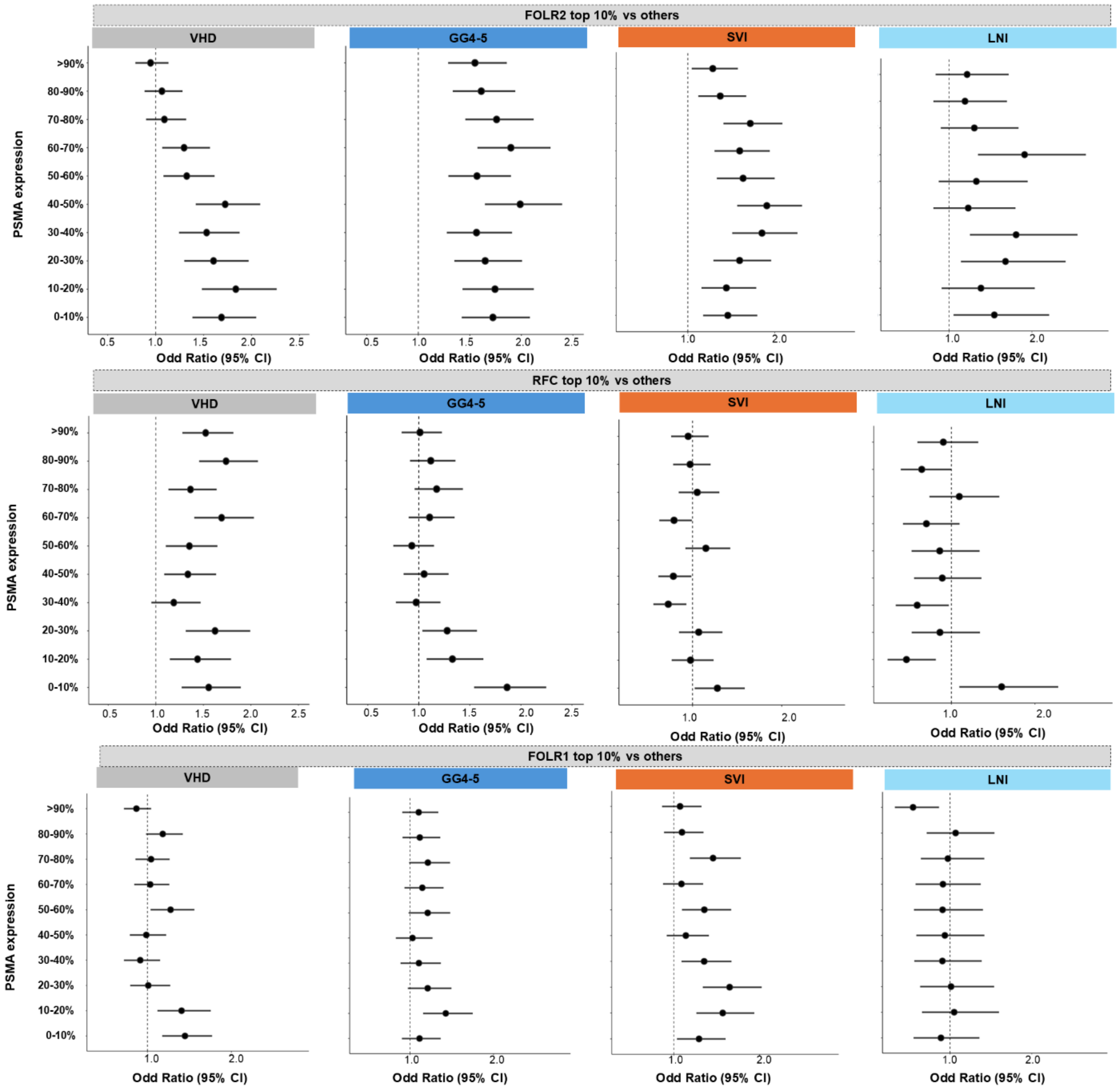

Forest plots present the odds ratios (95% CI) for very high Decipher (VHD) scores (>0.85), Gleason Grade Group (GG) 4–5 disease, lymph node invasion (LNI), and seminal vesicle invasion (SVI) across deciles of FOLH1 (PSMA) expression, comparing tumors with high (top 10%) FOLR2 (FR-β), SLC19A1 (RFC), and FOLR1 (FR-α) expression to all samples.

Figure S5. Association of Varying PSMA, FOLR1/2, and RFC Gene Expression with Percentage of Neuroendocrine (NE)-like Cancers

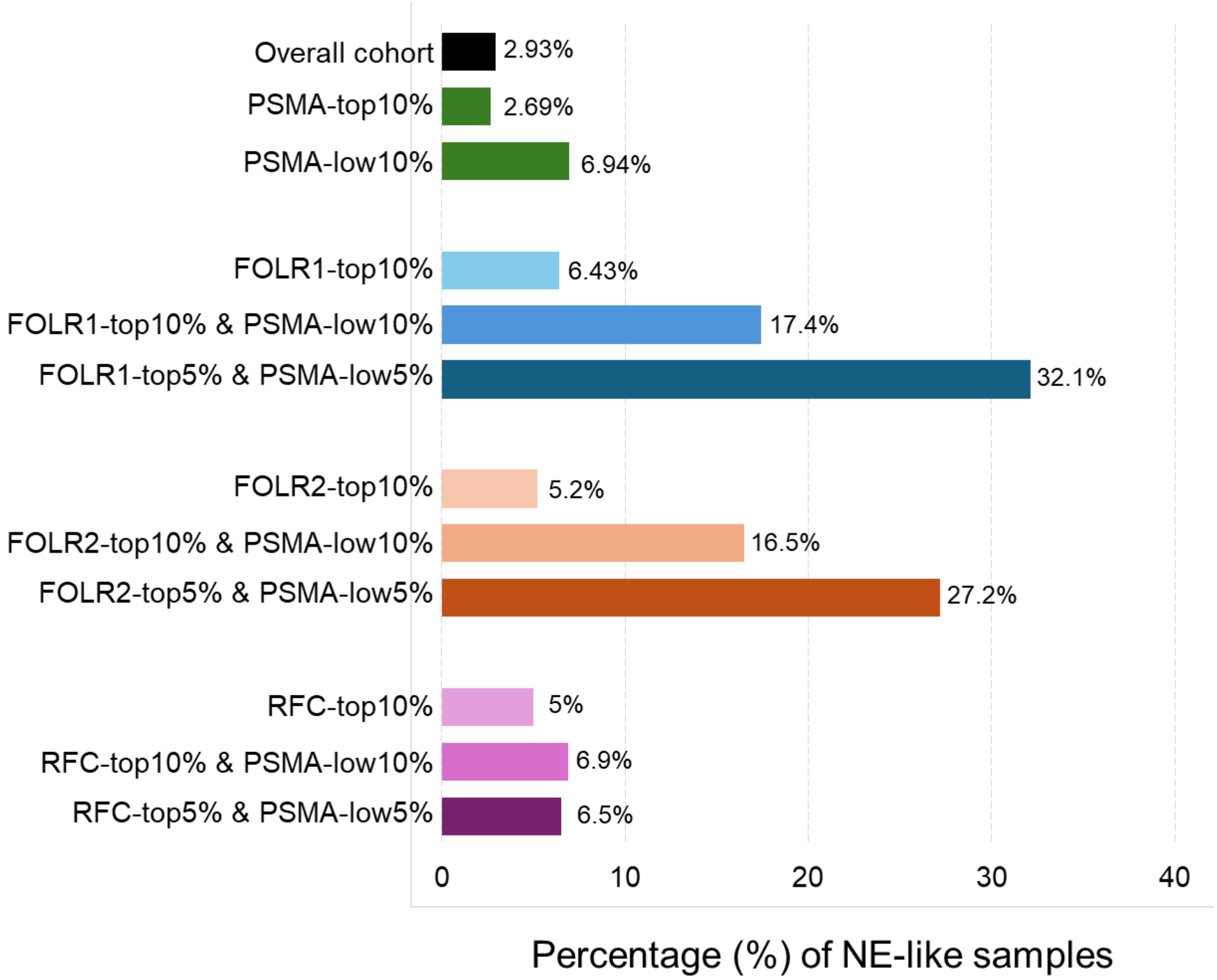

Supplement: Supplementary file 1 — Figure S1. Comparison of Overall Expression of PSMA and Folate‐Related Genes. Ridge density distribution plots display the expression patterns of PSMA (FOLH1), FOLR2 (FR‐β), RFC (SLC19A1), and FOLR1 (FR‐α) across all samples. Colour‐gradient maps illustrate relative gene expression intensity, ranging from low (purple) to high (yellow). PSMA (FOLH1) demonstrated the highest overall expression within the cohort, while FOLR2 (FR‐β) represented the predominant folate‐receptor subtype. B. Ridge density plot focused on FOLR1 and FOLR2 showing FOLR2 has higher expression. Figure S2. Evaluating Correlation Between Expression of Folate‐Related Genes Scatter‐density plots illustrate pairwise correlations between each of the folate‐transporter genes themselves – including FOLR2 (FR‐β), FOLR1 (FR‐α), and SLC19A1 (RFC) across all samples. Correlation coefficients ® are displayed in red, highlighting weak to moderate positive correlations between each of the folate transporter genes. Figure S3. Evaluating Percentage of Aggressive Prostate Cancer Features by Increasing PSMA, FOLR2, FOLR1 and RFC Expression Stratified by 5% Increments. Distribution plots depict the relationship between increasing FOLH1 (PSMA), FOLR2 (FR‐β), SLC19A1 (RFC), FOLR1 (FR‐α) expression and markers of aggressive disease, including Very High Decipher (VHD) scores and Gleason Grade Group (GG) 4–5 disease. Figure S4. Association of High FOLR2, RFC, FOLR1 expression (Top 10%) with Aggressive Prostate Cancer Features Across Increasing Ranges of PSMA Expression Forest plots present the odds ratios (95% CI) for very high Decipher (VHD) scores (>0.85), Gleason Grade Group (GG) 4–5 disease, lymph node invasion (LNI), and seminal vesicle invasion (SVI) across deciles of FOLH1 (PSMA) expression, comparing tumours with high (top 10%) FOLR2 (FR‐β), SLC19A1 (RFC), and FOLR1 (FR‐α) expression to all samples. Figure S5. Association of Varying PSMA, FOLR1/2, and RFC Gene Expression with Percentage of Neuroendocrine (NE)‐li [file BCO2-7-e70223-s001.pdf]
